# Integrating ctDNA-based *ESR1*m monitoring into first-line care for ER+, HER2− advanced breast cancer: a consensus workflow

**DOI:** 10.1093/oncolo/oyag411

**Published:** 2026-10-16

**Authors:** Yara Abdou, Amber Cipriani, Trevor Feinstein, Ryan Haumschild, Mabel Mardones, Joanne Mortimer, Eric Vail

**Affiliations:** Lineberger Comprehensive Cancer Center, UNC School of Medicine, University of North Carolina at Chapel Hill, Chapel Hill, NC 27599, United States; Department of Pharmacy, UNC Health Medical Center, Chapel Hill, NC 27514, United States; UNC Eshelman School of Pharmacy, University of North Carolina at Chapel Hill, Chapel Hill, NC 27599, United States; Piedmont Cancer Institute, Fayetteville, GA 30214, United States; Winship Cancer Institute, Emory University, Atlanta, GA 30322, United States; Sarah Cannon Research Institute, Denver, CO 80218, United States; Rocky Mountain Cancer Centers, Aurora, CO 80012, United States; Duarte Cancer Center, Duarte, CA 91010, United States; Cedars-Sinai Medical Center, Los Angeles, CA 90048, United States

**Keywords:** *ESR1*, ER+ HER2– advanced breast cancer, ctDNA monitoring, first line

## Abstract

Circulating tumor DNA (ctDNA) is an emerging tool in precision oncology that enables noninvasive tumor profiling, detection of minimal residual disease, and treatment monitoring. Furthermore, routine ctDNA analyses offer the potential to support more personalized and dynamic cancer care. In advanced breast cancer (aBC), ctDNA-based monitoring has been investigated across multiple studies as a tool for detecting emerging resistance mechanisms, predicting treatment responses and informing biomarker-guided treatment decisions. One example, investigated in the phase III PADA-1 and SERENA-6 trials, is monitoring for emergent estrogen receptor alpha mutations (*ESR1*m) during first-line endocrine therapy in estrogen-receptor positive, human epidermal growth factor receptor 2-negative (ER+, HER2–) aBC. While not yet widely adopted in clinical practice, broader implementation of routine ctDNA-based monitoring for *ESR1*m is anticipated and will require standardized diagnostic workflows, encompassing both institutional operational processes and patient-centered scheduling pathways. Insights and recommendations were gathered from multidisciplinary experts across academic and community practices in the United States regarding the feasibility, barriers, and operational requirements for implementing *ESR1*m monitoring during first-line treatment of aBC. Here, we present practical, experience-informed considerations, supplemented by an opinion-based consensus workflow model designed to provide clinicians with a flexible framework for implementing ctDNA-based *ESR1*m monitoring during first-line care for ER+, HER2– aBC.

## Introduction

Circulating tumor DNA (ctDNA) is an emerging noninvasive precision oncology tool, expected to significantly guide tumor profiling, enable disease monitoring and personalize cancer care.^1^ Within advanced breast cancer (aBC), ctDNA-based monitoring could help detect emerging resistance mechanisms, predict treatment responses, and guide treatment decisions.^2–5^ For estrogen-receptor positive, human epidermal growth factor receptor 2-negative (ER+, HER2–) aBC, ctDNA-based monitoring of *ESR1* mutations (*ESR1*m), the most common mechanism of acquired endocrine resistance, has been investigated.^4–8^

*ESR1* encodes estrogen receptor alpha (ERα), and acquired activating *ESR1*m predominantly occur within the ligand-binding domain (LBD) (Figure S1, see online supplementary material for a color version of this figure).^8^ In contrast to alterations in other pathways implicated in endocrine resistance (eg, PI3K/AKT/mTOR), *ESR1*m prevalence is low at diagnosis (∼5%), but frequently emerges under the selective pressure of endocrine therapy (ET), particularly aromatase inhibitor (AI)-based estrogen deprivation.^6–9^ Recurrent LBD mutations, such as D538G, Y537N, Y537S, and E380Q, contribute to endocrine resistance by stabilizing ERα in an active conformation, enabling ligand-independent ER transcriptional activity (Figure S1, see online supplementary material for a color version of this figure).^8^^,^^9^ The structural and functional heterogeneity of *ESR1*m provide a rationale for therapies that directly target ERα, including selective estrogen receptor degraders (SERDs).^8^^,^^9^ It should be noted that certain variants, including *ESR1* fusions, are not considered actionable, with no clinical trials or biologic rationale to support their sensitivity to ER-targeted therapies.^8^

Given the dynamic and treatment-emergent nature of *ESR1*m, routine testing during first-line ER+, HER2– aBC treatment may provide insight into resistance and tumor heterogeneity.^6^^,^^9^^,^^10^ However, guidelines currently vary in their recommendations for *ESR1*m testing at disease progression versus routine testing in the absence of disease progression (Table S1).^11^^,^^12^ The phase III PADA-1 and SERENA-6 trials investigated ctDNA-based routine monitoring for emergent *ESR1*m during first-line ER+, HER2– aBC care.^4^^,^^5^

PADA-1, although not registrational, demonstrated a progression-free survival (PFS) benefit with an early switch from first-line AI plus palbociclib to fulvestrant plus palbociclib after detection of rising *ESR1*m by serial targeted multiplex droplet digital polymerase chain reaction testing prior to radiographic progression.^4^ SERENA-6 incorporated routine ctDNA-based monitoring for *ESR1*m emergence every 2-3 months using the broader next-generation sequencing-based Guardant360 CDx assay in patients receiving ≥6 months of first-line AI plus cyclin-dependent kinase 4/6 inhibitor (CDK4/6i).^5^^,^^10^ Detecting emergent *ESR1*m in the absence of clinical or radiologic progression triggered a switch to the SERD and complete ER antagonist, camizestrant while continuing CDK4/6i, resulting in improved PFS maintained over longer follow-up, significantly improved PFS2, prolonged chemotherapy/antibody-drug conjugate-free survival and preserved quality of life.^5^^,^^13^^,^^14^

While camizestrant is approved in over 30 countries, including the United States, European Union, Japan, and the United Kingdom,^15–18^ the regulatory status alone does not establish the appropriate role for ctDNA surveillance in routine care. As camizestrant is now incorporated into NCCN Clinical Practice Guidelines in Oncology (NCCN Guidelines),^12^ broader implementation of ctDNA-based monitoring for *ESR1*m in routine clinical practice will require standardized diagnostic protocols, clear treatment pathways and streamlined institutional workflows.^19^ Implementation decisions should balance evidence strength and maturity, reimbursement and financial impact, ctDNA assay selection and performance, turnaround time, operational requirements, and equitable patient access.^19–21^

To inform this framework, roundtable discussions were conducted with fifteen precision medicine experts, including medical oncologists, pharmacists, pathologists, and nurse practitioners, representing fourteen different academic and community practices across the United States. Discussions focused on feasibility, operational barriers, and practical considerations for potential implementation of ctDNA-based *ESR1*m monitoring during first-line ET across diverse practices. Experts’ guidance was captured through thematic summaries, navigating differences by practice setting and professional role, and enriched by the authors’ clinical experience. The resulting opinion-based workflow can be adapted to local resources and standard operating procedures, and revisited as evidence, reimbursement landscape, and operational capabilities evolve. It is not intended to prescribe a uniform implementation model across settings, rather to outline options for institutions with differing levels of molecular pathology support, electronic medical record (EMR) integration, staffing, and sample-collection capabilities. Resulting opinion-based considerations are summarized and integrated into a step-by-step consensus workflow model for centers electing to implement this strategy in an appropriate clinical context (Figure 1).

**Figure 1.**
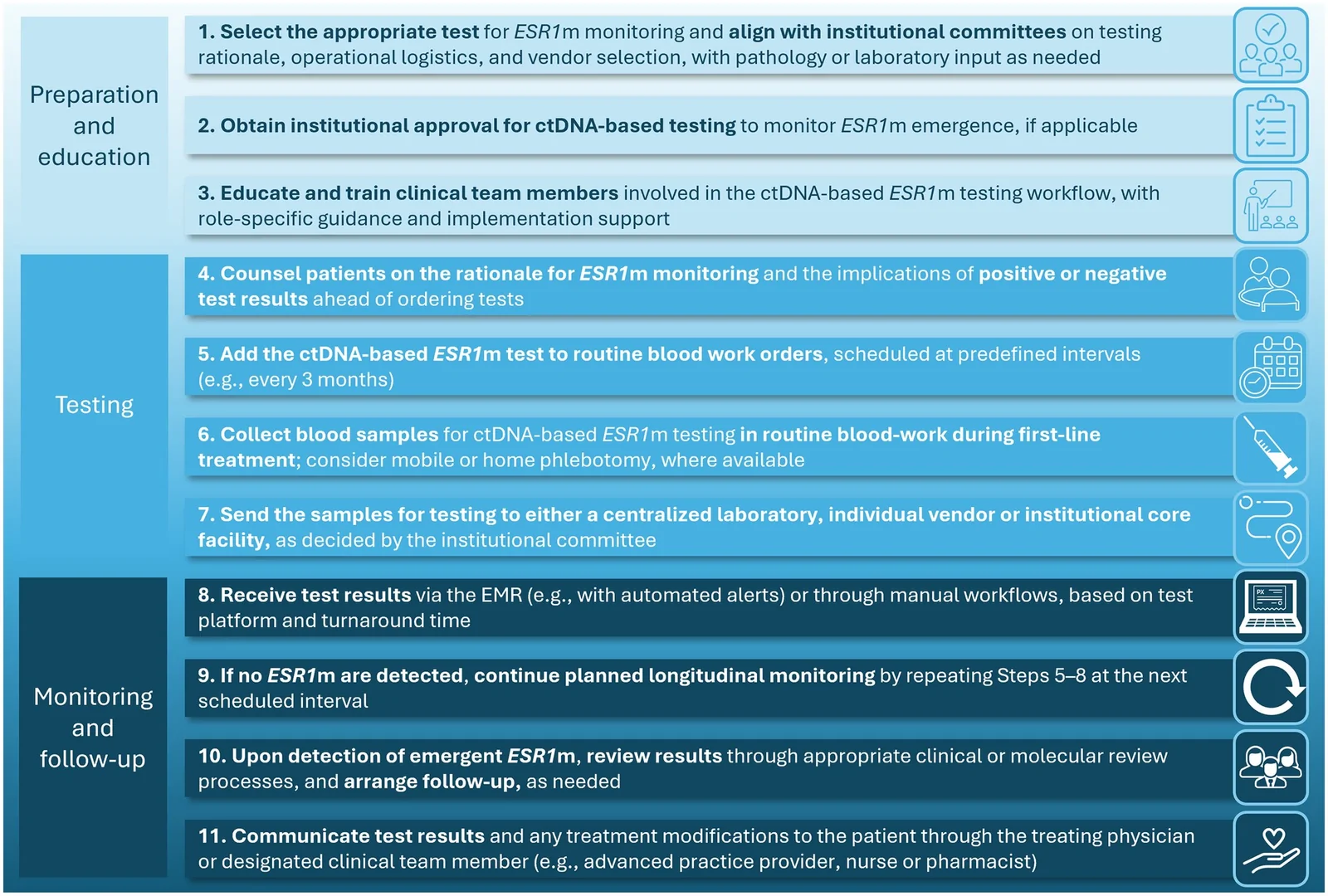
Practical workflow for implementing ctDNA-based *ESR1*m monitoring during first-line ET once the value of *ESR1* monitoring is established by regulatory bodies. This workflow outlines key operational steps from appropriate test selection and ordering for routine ctDNA-based *ESR1*m monitoring and clinical follow-up, as appropriate. It is intended to be adapted to local institutional standard operating procedures and practice settings, and to undergo periodic review as clinical evidence, regulatory status, and operational capabilities evolve.

## Using institutional committees to support test ordering

Processes for adopting treatment-related biomarker tests vary across practices, driven partly by differing testing platforms and technologies. In academic centers, disease-specific molecular precision medicine teams guide test selection and adoption, whereas in community practices, decisions are overseen by molecular programs, clinical committees, or laboratory leadership. Multidisciplinary executive committees support decision-making by evaluating clinical utility, operational feasibility, and cost–benefit considerations. Before ctDNA-based *ESR1*m monitoring is adopted, these committees should consider the evidence maturity and limitations, guideline alignment, reimbursement, financial impact, and potential implications for equitable access (Figure 2). This process is intended to support institution-specific evaluation of appropriate testing implementation and does not imply adoption across all practices.

**Figure 2.**
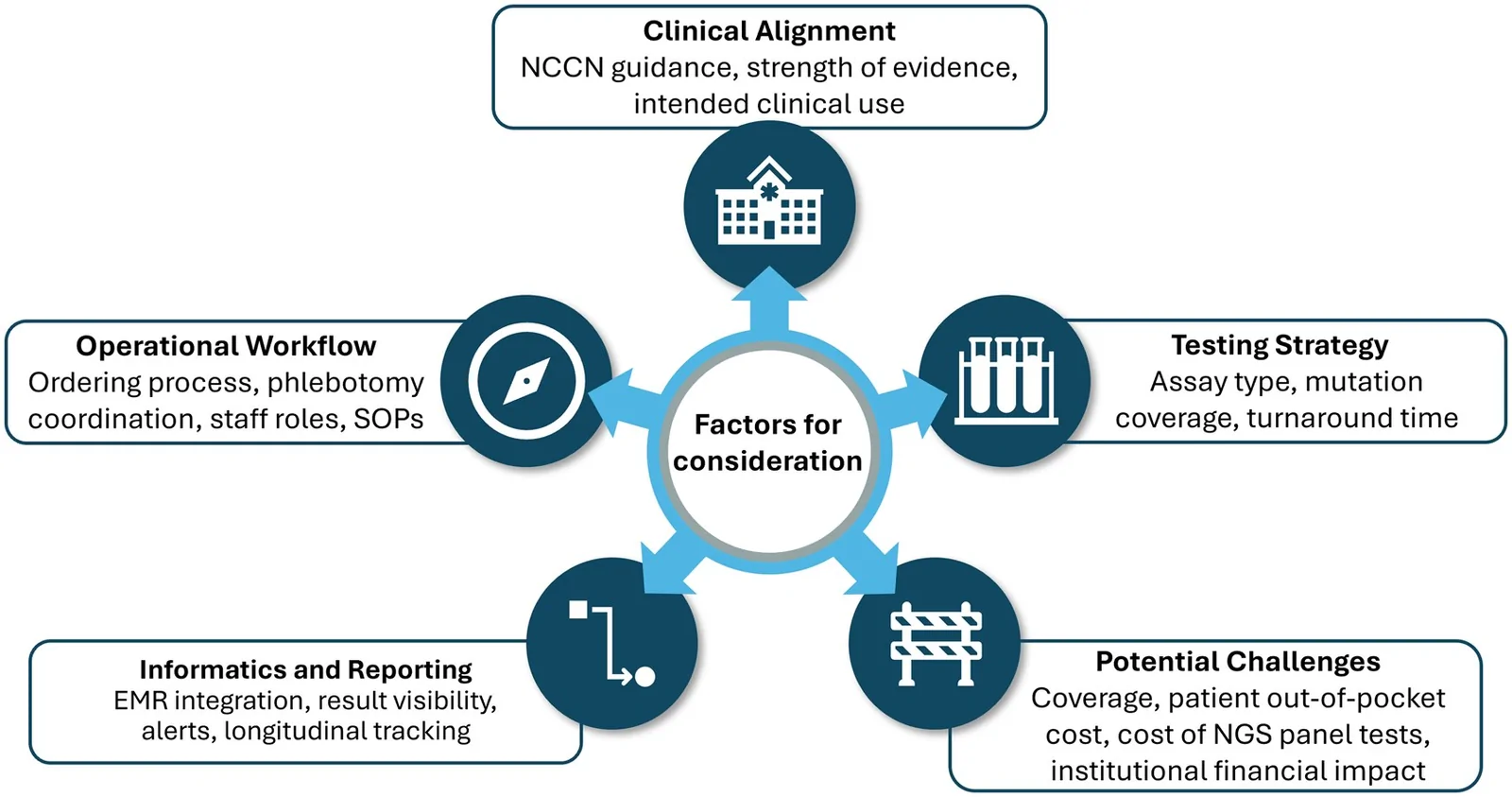
Key considerations for practices prior to implementing routine ctDNA-based *ESR1*m monitoring. Implementation decisions should account for alignment on clinical utility, selection of appropriate test with cost considerations (both for patients and institutions as applicable), testing strategy, operational workflow, informatics infrastructure, and cost considerations, and may vary based on institutional resources and practice setting.

### Roles of clinicians and care teams

The decision to order blood-based actionable biomarker testing mostly rests with the treating oncologist, with pathologists supporting test selection. For routine *ESR1*m monitoring, decisions about testing and result interpretation should be made within the context of guideline recommendations, local institutional policies, and evolving evidence. Oncology pharmacists and other advanced practice providers may play strategic and operational roles in implementing ctDNA-based testing, including developing treatment pathways and ordering tests under collaborative practice agreements.

Clear delineation of roles can facilitate efficient ordering, care team and patient result review, and downstream treatment decisions. Coordination of additional staff can ensure efficient sample collection. Designating a local clinical champion to oversee implementation, address operational challenges, educate clinical staff, promote consistent use of the workflow and periodically reassess the workflow as evidence, reimbursement, and local operational capabilities evolve may also support adoption.

## EMR integration of the ctDNA-based *ESR1*m test

Many institutions order ctDNA testing through EMRs, while others use manual processes. If routine *ESR1*m monitoring becomes institutionally approved, ordering ctDNA-based testing can be incorporated into routine order sets, and if possible, completed within the EMR.

EMR integration can streamline test ordering and reporting, enabling timely access to support treatment decisions. Where feasible, partnering with vendors that support bidirectional, automated EMR workflows may minimize operational burden. *ESR1*m results should be delivered through the EMR with discrete results, standardized reports, consistent nomenclature, and actionable alerts to support efficient review and longitudinal tracking. If EMR integration is unavailable, portal-based or manual workflows may require additional staff training. Training physicians, nurses, pharmacists, and support staff on EMR-based ordering, reminder systems, and result interpretation can further support successful and sustainable implementation.

## Minimizing the number of patient visits

### Scheduling *ESR1*m testing with routine blood work during first-line therapy

In SERENA-6, samples for blood work and ctDNA-based *ESR1*m assessments were obtained at routine visits approximately 3 weeks prior to follow-up appointments to discuss the results.

If institutions elect to use *ESR1*m monitoring, real-world operational challenges may present when coordinating imaging with blood draws for ctDNA testing, particularly in community settings. If additional clinic visits are required, the logistical burden should not be underestimated, and visit frequency minimized whenever possible. Where clinically appropriate and consistent with the selected testing strategy, ctDNA-based *ESR1*m monitoring in first-line treatment could be incorporated into routine blood work, typically requiring 1 or 2 additional blood tubes at scheduled visits. However, the testing interval may not be uniform across settings and should reflect available evidence, guideline recommendations, and local protocols.

Mobile phlebotomy or home blood collection may reduce scheduling pressures and patient burden, however, their availability, reliability, cost, and potential effect on access should be considered before incorporation into a workflow. Furthermore, reimbursement-related administrative workload could be mitigated through advance payer notification and procedures that outline coverage considerations.

Upon detection of emergent *ESR1*m, clinical progression status should be investigated to determine the next action. Routine imaging schedules should be maintained according to standard practice.

### Counseling patients on the rationale of *ESR1*m testing during first-line therapy

In routine practice, patients can sometimes access their results before discussion with their clinical team. The effect of routine ctDNA-based *ESR1*m monitoring on patient anxiety and other patient-centered outcomes remains an important focus area. Although patient-reported outcomes from SERENA-6 did not demonstrate increased anxiety, the potential psychological impact of repeated testing or result disclosure warrant further investigation.^13^^,^^22^

Before testing, clinicians should provide balanced counseling regarding the purpose of *ESR1*m monitoring in first-line care, its clinical utility, and long-term benefit to prevent unnecessary visits and reduce anxiety. Patients should understand that a positive result for emergent *ESR1*m may reflect molecular evolution before radiographic progression. However, it does not necessarily entail immediate changes in symptoms, prognosis, or treatment, while a negative result does not exclude future *ESR*1m emergence or replace routine clinical and radiographic assessment.

Additionally, patients should receive balanced, upfront information about the investigational or evolving nature of the testing approach, lack of consensus on implementation, potential benefits and uncertainties, and alternative approaches to support informed decision-making. Furthermore, discussions should address potential costs and reimbursement, and how results may influence management within the local care pathway.

If emergent *ESR1*m are detected, the patient’s treatment plan may be modified and individualized to the patient’s overall clinical status, imaging findings, prior therapies, treatment options, preferences and the applicable regulatory, guideline, and institutional context, rather than the molecular result alone. Oncology pharmacists and advanced practice providers may support communication of results, treatment switch coordination when indicated, and ongoing monitoring and scheduling. If *ESR1*m are not detected, negative results should be communicated via clear, secure, automated messages on patient portals or messaging systems and should avoid implying that a negative result confirms treatment benefit or absence of disease progression. Patient education on the utility of testing, whatever the result, may help ensure routine monitoring continues.

## Remaining questions and future directions

Routine ctDNA-based *ESR1*m monitoring during first-line therapy is evolving, with further evidence needed to establish clinical utility, long-term benefit, cost-effectiveness, feasibility, reimbursement, and equitable implementation. The optimal testing assay and interval, and management of discordant molecular, clinical, and radiographic findings are yet to be defined. If incorporated into practice, prospective real-world studies on implementation across diverse practices could determine the feasibility and equitability of implementing standardized workflows, focusing on testing uptake, turnaround time, patient and care team burden, and disparities in access and outcomes.

## Conclusions

Routine *ESR1*m monitoring may influence treatment decisions and requires standardized workflows to minimize burden on clinical teams and patients.^19^ This publication provides an insight-driven, practical framework to support institutional evaluation and operationalization of ctDNA-based *ESR1*m monitoring during first-line ET, where appropriate. Workflows should be adaptable to real-world operational variation and refined as clinical evidence, regulatory status, guidelines, reimbursement, and diagnostic capabilities evolve.

## Supplementary Material

oyag411_Supplementary_Data

## Data Availability

No new data were generated or analyzed in support of this research.
