# Supplementary material for: Integrating ctDNA-based ESR1m monitoring into first-line care for ER+, HER2− advanced breast cancer: a consensus workflow
Source: Oncologist. 2026 Oct 16;31(11):oyag411. doi: 10.1093/oncolo/oyag411 (PMC13693363; doi:10.1093/oncolo/oyag411)
Supplement: Supplementary file 1 [file oyag411_supplementary_data.zip › Supplementary Figure.docx]

**Supplementary material - Figure**

**Figure S1.** Activating *ESR1* mutations cluster predominantly within the ERα ligand-binding domain. A) *ESR1* structure and recurring activating mutations; B) Emergence of *ESR1*m and mechanisms of endocrine resistance.

Abbreviations:

1L, first-line; AI, aromatase inhibitor; CDK4/6i, cyclin-dependent kinase 4/6 inhibitor; ctDNA, circulating tumor DNA; ERα, estrogen receptor alpha; ESR1, estrogen receptor 1; ET, endocrine therapy.

References:

1. Grinshpun A, Chen V, Sandusky ZM, et al. ESR1 activating mutations: From structure to clinical application. *Biochimica et Biophysica Acta (BBA) – Reviews on Cancer.* 2023;1878(1):188830. <https://doi.org/10.1016/j.bbcan.2022.188830>
2. Bhave MA, Quintanilha JCF, Tukachinsky H, et al. Comprehensive genomic profiling of ESR1, PIK3CA, AKT1, and PTEN in HR(+) HER2(−) metastatic breast cancer: prevalence along treatment course and predictive value for endocrine therapy resistance in real-world practice. *Breast Cancer Res Treat*. 2024;207(3):599–609. <https://doi.org/10.1007/s10549-024-07376-w>
3. Chaudhary N, Chibly AM, Collier A, et al. CDK4/6i-treated HR+/HER2- breast cancer tumors show higher ESR1 mutation prevalence and more altered genomic landscape. *NPJ Breast Cancer*. 2024;10(1):15. <https://doi.org/10.1038/s41523-024-00617-7>
4. Brett JO, Spring LM, Barda A, et al. ESR1 mutation as an emerging clinical biomarker in metastatic hormone receptor‑positive breast cancer. *Breast Cancer Res*. 2021;23(1):85. <https://doi.org/10.1186/s13058-021-01462-3>
5. Betz M, Massard V, Gilson P, et al. ESR1 Gene mutations and liquid biopsy in ER-positive breast cancers: a small step forward, a giant leap for personalization of endocrine therapy? *Cancers (Basel)*. 2023;15(21):5169. <https://doi.org/10.3390/cancers15215169>
6. Bidard FC, Park YH, Mayer E, et al. Emergence of ESR1 mutations (ESR1m) in ctDNA during first-line (1L) endocrine-based therapy in HR+/HER2− advanced breast cancer: findings from the SERENA-6 trial. *ESMO Open.* 2026;11(Suppl 4):107442
