# Supplementary material for: Integrating ctDNA-based ESR1m monitoring into first-line care for ER+, HER2− advanced breast cancer: a consensus workflow
Source: Oncologist. 2026 Oct 16;31(11):oyag411. doi: 10.1093/oncolo/oyag411 (PMC13693363; doi:10.1093/oncolo/oyag411)
Supplement: Supplementary file 1 [file oyag411_supplementary_data.zip › Supplementary Table.docx]

**Supplementary material - Table**

| **Guideline** | **Recommendation** | **Year** |
| --- | --- | --- |
| American Society of Clinical Oncology (ASCO)^1^ | Recommends routine testing for emergence of *ESR1*m at recurrence or progression on ET (with or without CDK4/6i). Testing with a CLIA-certified assay should be performed on blood or tissue obtained at the time of progression, as *ESR1*m develop in response to selection pressure during treatment and are typically undetectable in the primary tumor. Blood-based ctDNA is preferred owing to greater sensitivity | 2023 |
| NCCN Clinical Practice Guidelines in Oncology (NCCN Guidelines®) V7.2026^2^ | Assess for *ESR1* mutations at progression following prior lines of endocrine therapy (ctDNA preferred). If *ESR1* is negative and TF is low, consider reflex testing with tissue-based next-generation sequencing assay | 2026 |
|  | If *ESR1* mutation is detected (category 2B) in a patient on an AI + CDK4/6 inhibitor for at least 6 months, in the absence of radiologic progression, could consider switching to camizestrant and continue the same CDK 4/6 inhibitor (category 2B) |  |

**Table S1.** Current US-based guidelines for *ESR1*m testing in ER+, HER2– aBC.

Abbreviations:

aBC, advanced breast cancer; AI, aromatase inhibitor; ASCO, American Society for Clinical Oncology; CDK4/6(i), cyclin-dependent kinase 4/6 (inhibitor); CLIA, Clinical Laboratory Improvement Amendments; ctDNA, circulating tumor DNA; ER, estrogen receptor; *ESR1*m, estrogen receptor alpha mutation; ET, endocrine therapy; HER2, human epidermal growth factor receptor 2; NCCN, National Comprehensive Cancer Network; TF, tumor fraction.

References:

1. Burstein HJ, DeMichele A, Somerfield MR, et al. Testing for ESR1 Mutations to Guide Therapy for Hormone Receptor–Positive, Human Epidermal Growth Factor Receptor 2–Negative Metastatic Breast Cancer: ASCO Guideline Rapid Recommendation Update. *J Clin Oncol.* 2023;41:3423–3425. <https://doi.org/10.1200/JCO.23.00638>
2. Referenced with permission from the NCCN Clinical Practice Guidelines in Oncology (NCCN Guidelines®) for Breast Cancer V.7.2026. © National Comprehensive Cancer Network, Inc. 2026. All rights reserved. Accessed [October 01, 2026]. To view the most recent and complete version of the guideline, go online to NCCN.org. NCCN makes no warranties of any kind whatsoever regarding their content, use or application and disclaims any responsibility for their application or use in any way
